# Supplementary material for: Chromosomal genome assembly resolves drug resistance loci in the parasitic nematode Teladorsagia circumcincta
Source: PLoS Pathog. 2025 Feb 6;21(2):e1012820. doi: 10.1371/journal.ppat.1012820 (PMC11801625; doi:10.1371/journal.ppat.1012820)
Supplement: Attachment — Submitted filename: Review_PLoS Path_D-24-01567.docx [file ppat.1012820.s018.docx]

McIntyre and colleagues have produced a highly contiguous genome assembly for *Teladorsagia circumcincta*, a major pathogen in sheep and goat farming in temperate climates. This new assembly is comprised from five autosomal and one X-linked chromosomal-scale scaffold, has 22,948 annotated genes and is 96.3% complete. Leveraging these genomic resources, the authors conducted a genome-wide selection scan of new and previously published data generated through 3 distinct mapping approaches (pre vs post treatment comparisons, introgression mapping, and comparison between different isolates with known resistance profiles) and 2 different genotyping methods (pool-seq and ddRAD-seq of individual worms). These analyses identified genomic regions associated with drug resistance across multiple anthelmintic classes, including benzimidazole, levamisole, and ivermectin. Some of these regions contain known genes and genetic variants already implicated in anthelmintic resistance in *T. circumcincta* and phylogenetically close species, while others represent novel regions not previously linked to resistance associated genes. The improved genome has enabled a more accurate interpretation of genome-wide genetic variation, enhancing our understanding of the genetic basis of drug resistance in this important species. The resources generated by this study significantly advance the fields of veterinary parasitology and helminth genomics and will support future research efforts aimed at developing diagnostic markers and improving control strategies.

The importance of contiguous genome assembly for the accurate interpretation of genome-wide genetic data has already been well established by the authors and others in the parasite genomics field. Perhaps a more nuanced and critical takeaway from this work is that, while a high-quality reference assembly is important, sufficiently resolving drug resistance loci to identify genetic markers remains a significant challenge. Although the FST peaks were linked to known resistance genes in the region, it appears that the resolution of the association signal was not sufficient to *de novo* identify any of the established, let alone novel, anthelmintic resistance genes from the genomic data. This observation does not necessarily diminish the value of the present work but rather highlights an opportunity to explore the various other factors (such as differences in mapping approaches, sampling, and genotyping methods) that influence the resolution of genome-wide studies aimed at identifying resistance loci that can serve as diagnostic markers. A more in-depth discussion of these factors would offer valuable insights and serve as guidelines for future studies and experimental designs in *T. circumcincta* and other related species.

Line 45: “573 Mb assembly (N50 = 84 Mb, n = 1,286)”. The "n" represents the total number of scaffolds in the assembly, not the N50 number. To clearly convey this, please consider revising it to “scaffold n = 1,286.”

Line 159: “genome assembly for the UK-derived MTci2 isolate of *T. circumcincta* (tci2_wsi3.0; Table 1)”. Please provide the assembly accession here or elsewhere in the paper.

Line 173: “gene content (Table 1, Supplementary Table 4)”, Based on information in Supplementary Table 4, two different versions of the Tci2 assembly (Tci2_wsi2.4 and Tci2_wsi3.0) were used for the BUSCO analysis, please use only the version described in the paper. BUSCO proteome results should be reported based on the longest isoform per gene for *H. contortus* and *C. elegans*. Compleasm results are likely more accurate than BUSCO ( and were included in Supplementary Table 4), I suggest reporting the compleasm results in Table 1.

Line 174: “repetitive content (Supplementary Table 5)”. Assembly names are not consistent across the text, tables, figures, and supplementary material.

Line 244: “Comparison of genetic differentiation between pre- and post-treatment on Farm 1 (Figure 2A; top panel) and Farm 2 (Figure 2A; middle panel) both revealed...” The figure legend indicates that the dashed lines in Figure 2A represent a genome-wide level of significance (FST = 0.0339). This value was determined based on the mean + 5 standard deviations of the genome-wide FST calculated from technical replicates of the Farm 2 pre-treatment group (Supplementary Figure 2).

“DNA from Farm 2 samples was split into two sub-samples (“A” and “B” samples), from which individual libraries were generated and sequencing performed.” It is not clear when the samples were pooled. Did you pool the L3 larvae prior to DNA extraction, or did you pool equimolar DNA prepared separately from each individual larvae? Did the "A" and "B" samples come from the same group of larvae, or from two distinct groups of larvae sampled from the same population? To empirically determine the outlier threshold using replicates, one would need distinct pools of larvae sampled from the same population. Additionally, the measured variance of FST depends on both, number of individuals in the pool and the total sequencing depth of each pool, and the extent of deviation from the equimolar assumption between individuals. These variables need to be kept constant across all pools included in the analysis to use a single threshold value across multiple comparisons between different pools. Essential information about the experimental design (e.g., number of individuals, sequencing depth for each pool) is missing, making it difficult to assess whether the FST distribution in the replicates adequately represents the null distribution expected in the absence of selection.

Line 246: “peak of differentiation”. Starting from this paragraph, the distribution of FST outlier regions (peaks) identified from various mapping experiments are described. However, these peaks are not clearly and consistently defined. The genome-wide significance level for outlier region was defined as 0.0339 for the farm data and as the mean + 3 standard deviations for the strain comparisons. However, no threshold was applied to the peaks in the Choi data. Using a common threshold definition across all datasets would make the comparison of peaks across datasets easier and may more clearly highlight the distinct features of each dataset using different mapping approaches. In addition, not all sliding windows with high FST values above the significance threshold were considered to represent peaks, to account for noise in the data. However, it was not clearly defined how many adjacent or closely spaced outlier windows were required to consider the region as a peak. This lack of clear definition resulted in suggestive language throughout the text, such as “a possible peak,” “may be shared by,” “potential peak,” “suspected peak,” etc. After defining the outlier regions (peaks), they could perhaps be labelled in the figure and referenced in the text. This would greatly enhance readability, given the number of comparisons and peaks discussed in this and the following paragraphs. Finally, please consider including the FST values, their Z-scores, the number of variant sites used to calculate the mean FST, and the read coverage breadth for the individual sliding windows in the supplementary material.

Line 247: “suggesting a common genetic response to ivermectin treatment.” Cochran-Mantel-Haenszel (CMH) test can be performed on the combined Farm1/Farm2 dataset to identify the common genetic response to IVM treatment and differentiate it from farm-specific variation in a more statistically principled manner.

Line 251: “Comparison of the post-treatment populations from Farm 1 and Farm 2 revealed a more complex history of genetic change and likely drug-mediated selection”. Distinguishing the effects of drift and selection in between-farm comparisons is challenging. However, comparing the pre-treatment populations from Farm 1 and Farm 2 could provide a clearer insight into historical genetic changes, as opposed to the post-treatment population comparisons presented in the paper, which are confounded by the direct effects of selection.

Line 297: “Comparison across all three panels reveals common peaks of differentiation”. CMH test could be useful for identifying and statistically supporting common genetic differences across multiple pairs of pools.

Line 320: Figure 2. The assembly name could be removed from the chromosome ID. The life cycle stage of the samples used in the Figure 2C comparisons was not provided.

Line 340: “We have compiled a list of candidate genes associated with individual drug classes and previously described variants associated with resistance (Supplementary Table 7).” Please indicate which of these candidate genes were contained within the FST peaks identified in this study and provide references for the previously described variants associated with resistance. The resistant allele frequencies and the statistical significance of the frequency differences (from the relevant pairwise comparisons) should be included.

Line 355: “we identify the F200Y allele at high frequency in all strongly resistant isolates, as well as the E198L variant”. Choi et al. (2017) reported that the F200Y and E198L variants likely occur only in trans on separate haplotypes in their study population from New Zealand. (minor comment, introduce earlier than the discussion section in the manuscript that the 2017 study is based on New Zealand strain). They concluded that the absence of haplotypes simultaneously harbouring both variants suggests that E198L can confer BZ resistance independently of F200Y and that beta-tubulin carrying both variants is either detrimental to organismal fitness or that the double mutation event (or recombination over an interval of <6 bp) required to produce a cis haplotype is so unlikely that it has not been observed. Have you investigated the haplotype structure around the F200Y and E198L variants in the UK samples, particularly in the Farm samples? Could further insights be drawn from these comparisons?

Choi et al. also noted that F200Y occurs on at least two distinct and diverse haplotype backgrounds, suggesting multiple independent origins of the variant allele. When multiple distinct haplotypes are under selection, soft sweep-like signatures may be observed around the loci. In these regions, individual SNP-level (FST or allele frequency) comparisons can provide stronger and more meaningful signals compared to sliding window analysis. For example, individual SNP-level analysis within the major ivermectin peak (Chr 5) may provide additional resolution to narrow down the candidate loci.

Pool-seq data enables the analysis of within-population genetic diversity, such as pi. Comparing pi between pre- and post-treatment samples can offer valuable insights into the extent of genetic diversity loss driven by selection and, consequently, the nature of that selection. Have you observed a pronounced decrease in genetic diversity within the identified FST peaks following treatment? These analyses could provide complementary evidence for the resistance loci, particularly in the novel regions.

Line 372: Figure 3. Please revise the X-axis title. "Sampling location" is appropriate only for the Farm samples. Include the statical significance of the frequency differences (e.g., Fisher’s exact test using allele counts).

Line 417: “A single peak on chromosome 3 (peak range: 15-20 Mb) found only in the MTci7:MTci2 comparison and not in the other ivermectin-resistant isolate comparisons is suggestive of a moxidectin-specific response.” As discussed in my previous comment (Line 246), because FST peaks are not clearly defined, it is difficult to determine whether the peak on Chr 3 (15-20 Mb) is truly a moxidectin-specific response region and whether there are other, potentially more significant peaks.

Line 438: “compared Fst from pooled sequencing (Figure 4A) with an independent experiment using ddRAD-seq”. It is not clear what is meant by “independent experiment.” Line 942 states, “These L3 were a subset of those included within the larger number pooled for whole-genome sequencing above.” Clarification is needed.

Line 439: “ddRAD-seq to assess linkage disequilibrium (LD)...”. LD decay analysis (Fig 4B) is likely sensitive to variations in missing genotype calls and allelic dropouts between the pre- and post-treatment populations. Have you confirmed that the differences in the decay patterns were not associated with any nonrandom genotype missingness in the ddRAD-seq dataset? Did you observe an FST peak in the ddRAD-seq dataset comparable to that from the pool-seq analysis? Was the density and distribution of ddRAD loci adequate for reliably measuring the rate of LD decay at a sufficiently high resolution? Because continuous line graphs were used, and decay distance at a single r^2 value was plotted, it is difficult to determine how robust and well-supported the signal is. An alternative approach that visualizes all genotyped locations and all pairwise LD values within the region (e.g., PMID: 33126247) may help readers better assess the significance of the LD variation between pre- and post-treatment populations.

Line 442: “consistent with a reduction in diversity associated with drug-mediated selection”. Sliding window analysis of pi using the pool-seq and/or ddRAD-seq data would show the reduction in diversity in the region.

Line 451: “variation in coverage was noted”. Coverage ratio variation (at a 100 kb window size resolution) in Fig 4C likely indicates copy number variation (CNV) within the FST peak. The maximum ratio (~1.5) could be an underestimation if the size of the CNV is relatively small compared to the window size. Using a dedicated CNV caller (e.g., PMID: 22302147) rather than relying on a heuristic approach would improve both the sensitivity and specificity of CNV detection and may identify resistance-associated CNVs.

Line 455: “These results collectively suggest that the locus under ivermectin selection lies between 35-38 Mb”. Conducting the CMH test across all pairwise comparisons that show differentiation signals across the Farm, Choi, and Strain datasets would reduce noise from individual comparisons and could significantly narrow down the regions under direct selection, distinguishing them from linked loci. This approach would highlight the long-term comparability of WGS data across multiple studies and experiments, and the value of the improved assembly, which enables more accurate re-analysis and integrated analysis with previously published data.

Line 480-489. These perhaps could be moved to the discussion section.

Line 504: Supplementary Figure 7. The figure suggests that there are genes likely displaying CNV in each pool-seq comparison. However, since the order of genes along the X-axis varies between comparisons, it is difficult to determine whether any genes other than pgp-9 show CNV across multiple comparisons. Was pgp-9 the only gene consistently detected across multiple comparisons?

Line 597: “We interpret these more complex signals...” The limited number of backcrosses (n=2) used to generate the RS3 introgressed line in Choi et al paper is likely the primary reason for the broader peak regions observed, rather than the severe bottlenecking that occurs during co-selection. Since sexual recombination occurs before drug treatment, the recombination itself is not directly impacted by bottleneck events. However, the “effective” recombination rate is influenced by the genetic divergence between the two parental lines in the crossing experiment, which can affect mapping resolution. Regardless of the severity of the bottlenecking during selection, as long as there is sufficient genetic divergence between the parental lines and the treatment does not kill all individuals during selection, it would still be possible to achieve high mapping resolution in the introgressed line, provided that a sufficient number of backcrosses are performed.

Line 645: “Finally, comparing pre and post-ivermectin treatment parasite populations has lowered the background genetic variation commonly observed for genetic comparisons of distinct isolates, allowing identification of a single locus of differentiation. In contrast, between isolate comparisons identified multiple peaks of differentiation which could not be confidently assigned to specific drug classes and may reflect non-drug-related selection and divergence between populations.” Both untreated and drug treated MTci5 samples were available for comparison. However, the treated MTci5 samples were not compared against the untreated sample. Were those comparisons done, and what was the reason for not including these comparisons in the paper?

Line 659: “Targeted, fine-scale mapping using samples from drug-specific experiments will likely yield further insight into the specific causal variants driving resistance”. What does “drug-specific experiments” mean in this context? Both Farm and Strain experiments used single drug selection, but it was not possible to prioritize a small number of candidate causal variants from these experiments.

A common limitation across the three datasets (Farm, Choi, and Strain) seems to be the difficulty in distinguishing causal loci under direct selection from a large number of linked variants, resulting in broader differentiation peaks. Sequencing individual worms is unlikely to fully address this issue. A paragraph detailing specific approaches and experimental design considerations that may help overcome this problem should be included.

Line 699: “sequenced on a single lane of Illumina”. The methods section frequently reports the number of lanes used in sequencing. While this is often done for historical reasons, it is not particularly relevant, as sequencing libraries across multiple lanes and runs on modern platforms do not introduce significant technical bias. Instead, please report the total number of gigabases (Gb) generated per sample, either in the relevant sections or in the supplementary material.

Line 864: “Protein sequence orthology between genome assemblies was assessed using Orthofinder” and line 867: “Functional annotations for the protein sequences were inferred using InterProScan”. Including a gene mapping between the WASHU and tci2 assemblies and providing the functional annotation results as supplementary material in the paper would enhance the usability of the new genome, particularly for researchers who may lack access to advanced computing resources.

Line 920: “All samples consisted of hundreds of adults or L4 worms, sampled before or after drug treatment, as described in Supplementary Table 2”. Please provide the approximate number of individuals pooled and the sequencing depth for each sample. This information is crucial for assessing the quality of the pool-seq experiment, as inadequate sampling can compromise the accuracy of the analysis. Additionally, please report the mapping rates for the pool-seq and RAD-seq data. Anomalous mapping rates may indicate the presence of contaminant DNA in the samples or, in species with high genetic diversity like T. circumcincta, could suggest limitations in read aligners when mapping highly divergent reads to the reference genome. These are important QC metrics that should be included in the Supplementary Table.
