# Supplementary material for: Chromosomal genome assembly resolves drug resistance loci in the parasitic nematode Teladorsagia circumcincta
Source: PLoS Pathog. 2025 Feb 6;21(2):e1012820. doi: 10.1371/journal.ppat.1012820 (PMC11801625; doi:10.1371/journal.ppat.1012820)
Supplement: Attachment — Submitted filename: R1_Response-to-reviewers.docx [file ppat.1012820.s019.docx]

Dear Editors,

We’d like to thank the Editorial team and the three reviewers for the carefully considered and overall positive appraisal of our manuscript. We appreciate the constructive comments and suggestions, which we have addressed in full below, that have helped us clarify the presentation of our data and conclusions. As requested, we have included a marked-up and clean version of the revised manuscript to highlight the changes made.

We hope these revisions are sufficient for you to reconsider publishing our manuscript in PLoS Pathogens.

Kind regards,

Jennifer McIntyre and Stephen R. Doyle

Co-corresponding authors, on behalf of all authors.

**Reviewer's Responses to Questions**

Reviewer #1: This is a clearly written, well presented, and scientifically rigorous description of the genomic analysis of anthelmintic resistance in Teladorsagia circumcincta. It should be accepted for publication in PLoS Pathogens. I have made some comments below, separated into those to which I think the authors should respond prior to final acceptance, and those that are of lesser importance.

As is clear from my comments, but I will state here as well, I am a co-author of the Choi et al. paper to which this paper refers extensively and from which the authors have extracted and re-analysed data. Some of my comments relate specifically to the original paper and the study it reported.

Reviewer #2: McIntyre and colleagues have produced a highly contiguous genome assembly for *Teladorsagia circumcincta*, a major pathogen in sheep and goat farming in temperate climates. This new assembly is comprised from five autosomal and one X-linked chromosomal-scale scaffold, has 22,948 annotated genes and is 96.3% complete. Leveraging these genomic resources, the authors conducted a genome-wide selection scan of new and previously published data generated through 3 distinct mapping approaches (pre vs post treatment comparisons, introgression mapping, and comparison between different isolates with known resistance profiles) and 2 different genotyping methods (pool-seq and ddRAD-seq of individual worms). These analyses identified genomic regions associated with drug resistance across multiple anthelmintic classes, including benzimidazole, levamisole, and ivermectin. Some of these regions contain known genes and genetic variants already implicated in anthelmintic resistance in *T. circumcincta* and phylogenetically close species, while others represent novel regions not previously linked to resistance associated genes. The improved genome has enabled a more accurate interpretation of genome-wide genetic variation, enhancing our understanding of the genetic basis of drug resistance in this important species. The resources generated by this study significantly advance the fields of veterinary parasitology and helminth genomics and will support future research efforts aimed at developing diagnostic markers and improving control strategies.

Reviewer #3: This manuscript describes a very much-improved Teladorsagia circumcincta reference genome constructed using PacBio long reads and Hi-C contact maps. This new resource enabled the mapping and further refinement of anthelmintic resistance loci, vastly improving results for this species, as the authors had done previously for H. contortus. This manuscript describes a much-needed community resource.

**RESPONSE:**

We appreciate the very positive appraisal of our manuscript from all three reviewers and their constructive comments and suggestions. We have addressed their comments and believe the changes have improved the manuscript.

**Part II – Major Issues: Key Experiments Required for Acceptance**

Reviewer #1:

Lines 345-68 & Fig 2: BZ resistance. An intriguing feature of the Choi et al. analyses was the absence of tubulin isotype 1 E198L / F200Y and double homozygotes and the consequent strong departure from Hardy-Weinberg equilibrium at this locus. The authors speculated that the this implied that the resistance alleles in heterozygotes were always in trans- rather than cis- on the basis that individuals homozygous for both resistance alleles were unviable. The highly fragmented nature of the Choi et al. assembly did not, however, permit testing of this hypothesis through haplotype reconstruction. This manuscript include very extensive PacBio sequencing, including sequencing from DNA made up of large pools (assuming that the 125K sheathed L3 referred to in Supp Table 2, for example, refers to 125,000 individuals, or 250,000 haploid genomes, per sequencing pool). Each PacBio read is, in effect, a haplotype. The inter-strain comparisons and GWAS are all based on measures that use allele frequency from genotyping of bi-allelic SNP loci. Could these authors please consider the value of considering haplotypes given that they have data that may permit haplotype imputation from the haplotype frequencies in the strain MTci2 PacBio data? It is worth noting that most of the genetic variation between individuals is haplotype variation generated by recombination, and it is on haplotypes rather than individual alleles at single loci, that selection will operate.

**RESPONSE:**

Haplotypes are important, especially with multiple causal alleles within the same gene. We did not originally seek to clarify the haplotypes of beta-tubulin E198L / F200Y variants, as they were already clearly and elegantly described in the Choi et al. paper.

In response, we have calculated haplotype frequencies of SS, SR, RS, and RR in the mapped sequencing reads of all of our pooled datasets. In all comparisons, we found consistent results to Choi et al., i.e., there are no RR in cis-orientation on the same haplotype, and therefore, individuals with both R variants are heterozygous, i.e., only RS and SR haplotypes are found. In response to this comment and new analysis, we have updated the text as follows:

*“ Consistent with Choi et al., we did not find any haplotypes containing resistant alleles for both E198L and F200Y in any pooled sequencing dataset (Supplementary Table 8), further supporting the hypothesis that heterozygotes for both alleles are in trans- rather than cis- and that individuals homozygous for both resistance alleles are unviable* [*(Choi et al. 2017*](https://paperpile.com/c/SRKqLV/s0nnh)*).”*

It is an interesting idea to look at PacBio data for haplotypes due to the long read length. However, we only have PacBio data for the susceptible MTci2 strain, which has very low levels of the F200Y and no E198L, so there is nothing to compare here.

Lines 400 – 410: in reference to the comment above concerning the importance of haplotypes, I note that lines 400 – 410 touch on the importance of genetic linkage of loci that may be under selection i.e., an issue of haplotypes rather than single alleles at single loci. As with old-fashioned Mendelian genetic mapping by recombination, perhaps looking for the rare recombinant that separates linked alleles at two loci involved in different resistances (in this case, BZ vs LEV) may be the way to go?

**RESPONSE:**

This is a good idea for resolving alleles that are physically very close to each other. We speculated that if there were variants associated with levamisole resistance close to *beta-tubulin isotype-1*, we would not be able to separate them from the current data. However, we do not have individuals phenotyped for levamisole resistance without concurrent benzimidazole resistance, nor the proper experiment to look for recombination frequencies between variants.

However, in response to this comment, we have added a sentence in the discussion as follows:

*“therefore, in these studies, if a genetic signal of differentiation associated with unc-29 was present, it would not be distinguished from the established signal associated with benzimidazole resistance. To do so, recombination mapping to identify independently segregating variants using sequencing of single-worms, phenotyped for levamisole sensitivity, could help map physically close variants associated with different drug classes.”*

• Lines 491 – 510: Tcpgp-9 variation. This paragraph describes data consistent with a causal involvement of increased copy number of the Tcpgp-9 locus in resistance to ivermectin in some of the lines analysed. The authors propose that this is unlikely to be a major driver of ivermectin resistance on the grounds of “The relative height of the pgp-9 peak to other peaks…”. I take this to be a reference to an Fst peak height (there is no specific reference to a figure here; line 507). Could these authors please explain in more detail why there should be a direct linear correlation between the copy number of a specific locus and Fst? Particularly if, as seems plausible for copy number variation in response to recently applied drug selection, the copy number expansion is recent and there is little genetic variation amongst the duplicated copies. One could imagine a small degree of sequence variation (measured by Fst) despite recent 4 – 10 fold expansion of copy number (Fig 4G).

**RESPONSE:**

To clarify, we said:

*“The relative height of the pgp-9 peak to other peaks associated with ivermectin response is suggestive that pgp-9 variation is not the primary driver of ivermectin resistance in MTci5 and RS^3^ isolates but could be a strong candidate in MTci7. “*

I.e., *pgp-9* is not the “primary” driver of ivermectin resistance because other peaks were more prominent and frequent in ivermectin comparisons. Indeed, in our field data comparing pre- and post-ivermectin-treated offspring populations, we did not find a peak over *pgp-9* at all; such peaks were only present when comparing different isolates.

Whether peak height is a good proxy for strength or selection or some other degree of “importance” is a good question. In our experience with *Haemonchus*, we see some correlation between peak height and an implied importance with causal variants, particularly differentiating mode of inheritance (single vs multiple genes) and resistant variant frequency in a pool. In addition, as we have demonstrated in this study, peak height varied between comparisons, with the greatest Fst values present in the Sinbred:RS3 comparison and the lowest present in the pre- vs post-treatment field data. However, the relative height of different peaks within a comparison was informative, as was demonstrated in the reversal of the relative peak heights on Chromosome 1 and Chromosome 5 in the MTci5(BZ):MTci2 and MTci5(IVM):MTci2 comparisons. In a pooled sequencing context, if pgp-9 confers resistance, then you would expect a selective sweep resulting in a peak of differentiation between susceptible and post-treatment/resistant pools of individuals.

We agree that if copy number expansion is recent and has been in direct response to treatment, then within the expansion, there will be little variation. However, a peak is far broader and includes many non-causal variants in linkage - if those resistant individuals are reproducing and recombining within a genetically diverse population, a peak will still be observed despite less diversity at the causal locus. In that context, if individuals are more fit due to more copies of a gene, then we might expect a stronger sweep and, therefore, a more prominent peak. This depends on many factors, including when selection occurs, how much recombination has occurred, and the frequency of the resistant individuals in the population.

Lines around and preceding 578 – 579. The authors make this more complicated than it needs to be and are possibly inadvertently misleading the reader by referring to “these shared signals of selection” when trying to express the view that (a) the mechanisms of BZ and IVM resistance are distinct, (b) selection for BZ resistance preceded selection for IVM resistance so that (c) worms are frequently resistant to both classes of drug, and thus display two mechanistically and genetically distinct signals of selection. The challenge is to determine which signal(s) relate to which resistance. The authors would do well to point to the Choi et al. strategy of using genetic recombination and single drug challenge to separate the signals for each drug class. There was little “shared signal” between drug classes in the F3 recombinant generation of the Choi study. I agree that attempting to reconstruct selection history through haplotype analysis (lines 575-85) may be necessary given the impracticality of repeating the Choi et al. genetic experiment (which took a number of years to complete), but the authors might consider attempting to rewrite this section of the Discussion. As written, it does not clearly annunciate the two related, but separate, routes to solving the problem that it is difficult to assign a peak of genetic differentiation to a specific drug resistance in multi-resistant strains: the genetic solution presented by Choi et al. vs the theoretical solution of reconstructing the history of haplotype selection.

**RESPONSE:**

We agreed that “shared” is a little misleading. In response to this comment, we have changed “shared” to “co-occurring”.

In Choi et al., we acknowledge that an attempt was made to identify ivermectin-specific signals based on a drug-specific treatment. However, a limitation of that study was that only one drug was examined, and the genetics were assessed using RADseq. Therefore, there is a confounding problem with that comparison—it is difficult to correctly interpret the comparison between (i) poolseq with all drugs and (ii) RADseq with ivermectin.

To address this comment, we have rewritten this section of the discussion as recommended:

*“Our analyses of pre-and-post ivermectin selection on two UK farms revealed a common signature of ivermectin selection on chromosome 5, which we propose contains a novel driver of resistance. No other regions appeared obviously under selection by ivermectin, however, a comparison of the post-treatment groups revealed a more complex history of selection by anthelmintics. Consistent with the fact that both farms had high levels of phenotypic benzimidazole resistance (by FECRT), a strong signal was identified around the beta-tubulin isotype 1 locus. Mutations previously associated with benzimidazole resistance were also identified on each farm. Taken together, these suggest that benzimidazole resistance likely arose independently on each farm* [*(Redman et al. 2015)*](https://paperpile.com/c/SRKqLV/TZ9d8)*. Furthermore, the between-farm differentiation at this locus and just adjacent to the ivermectin-treatment-associated locus (from 37.5-40 Mb on chromosome 5) likely reflects that selection has occurred independently on different genetic backgrounds rather than appearing on one farm and spreading to others, consistent with previous work* [*(Redman et al. 2015)*](https://paperpile.com/c/SRKqLV/TZ9d8)*. The use of ivermectin for the treatment of ovine helminths is typically intercurrent with other anthelmintic classes, for example, as a subsequent response to the lack of efficacy of benzimidazoles due to rotation between drug classes or as part of seasonal targeting of parasites. In the UK, for instance, it is common to treat lambs for Nematodirus battus infection with benzimidazole in spring but to then prefer ivermectin later in the grazing season on the basis of perceived effectiveness* [*(Melville et al. 2021)*](https://paperpile.com/c/SRKqLV/txZcA)*. In some parts of the world, combination products containing more than one nematode anthelmintic are used. Therefore, globally, ivermectin resistance commonly appears on a background of benzimidazole resistance, and while the mechanisms of action are distinct between these two drug classes, these co-occurring signals of selection have the potential to confound the interpretation of resistance mechanisms. Careful study design can help to pull these co-occurring signals apart. For example, using a single anthelmintic and comparing pre and post-treatment samples from an admixed population, as shown here, can help. Further phenotyping of sample populations in vitro, with subsequent individual or pooled sequencing, may also serve to distinguish selection signals. Importantly, these experiments will only be likely to work if the parasite population has sub-populations that are sensitive to one of the anthelmintics (as shown here), as otherwise, if the population is made up of only dual-resistant individuals (no signal will be seen) or either dual-sensitive or dual-resistant individuals (both signals will be seen), the experiment is less likely to be successful. Understanding the genetic relatedness of haplotypes in these regions, particularly from a denser sampling of parasite populations, will inform how resistance evolves and spreads.”*

Line 609 onwards: the authors might consider adding text to address the following to this paragraph. **First, in reference to lines 609 – 11,** it is clear that to be useful in a routine diagnostic sense, identifying these conserved pathways to resistance is essential. Do the authors see any potential here to discuss how identifying these conserved pathways might lead to practical outcomes? If not, why not? Second, and related to the first, the authors conclude that there “resistance [to BZ and IVM] has evolved independently on diverse haplotypes”. I agree, but how likely is this to impede the development of diagnostic tools based on the shared resistance mechanism (the endpoint of selection) even if the genetic history of selection is unique in each resistant population? What are the practical consequences? The authors may respond by saying that the aim of their work is provide better tools to investigate the genetic basis of resistance but not to develop tools for genetic diagnosis of resistance. I remind them that the final sentence states clearly that better genome data will “inform more effective and sustainable control” by identifying “the specific causal variants driving resistance”. Those variants are known for tubulin isotype-1 but no diagnostic tools are in routine use. How will providing analogous information on IVM and/or LEV resistance help? Very non-molecular work such as that described by Leathwick and others on how to use anthelmintics to delay and perhaps even revert resistance has provided practical answers.

**RESPONSE:**

Identifying conserved signals of selection gives us growing confidence that similar genes, if not similar genetic variants, are under selection in independent populations. This is very good news regarding the practical application of genetics for diagnosing resistance. In response to this comment, we have expanded on the text in the discussion as follows:

*“This has implications for diagnostics; where the same SNP is involved across species, allele-specific techniques can be used to identify resistance* [*(A. Silvestre and Humbert 2000)*](https://paperpile.com/c/SRKqLV/hDAo)*. Even where several closely spaced SNPs may independently confer resistance, as in the case of the three SNPs in the beta-tubulin isotype 1 gene, amplicon sequencing can identify them* [*(Avramenko et al. 2019)*](https://paperpile.com/c/SRKqLV/yarv)*. For monepantel resistance, a variety of mutations, each independently likely to alter the function of mptl-1, have been identified in phenotypically resistant T. circumcincta populations* [*(Turnbull et al. 2019)*](https://paperpile.com/c/SRKqLV/EPzy)*, but the diagnostic utility of this information is less clear and may require an understanding of haplotypes of variants across a larger region. Nonetheless, in the research setting, the ability to monitor resistant genotypes in specific species via sequencing technologies will allow a more detailed approach to anthelmintic resistance risk and refugia studies* [*(Melville et al. 2020)*](https://paperpile.com/c/SRKqLV/VQYy)*, given the limitations of the FECRT, which necessitates the use of anthelmintics. Even without a causal mutation being identified, the ability to monitor for reduction in diversity/changes in haplotypes over a small region of the genome using these modern sequencing technologies will enable the study of more complex loci and resistance mechanisms.”*

We agree that there are no molecular diagnostic tools in routine use, which is a shame, given we have robust variants that could be used now to diagnose and monitor resistance. However, we are also confident that this situation is beginning to change. This is evidenced by a recent surge in the use of molecular tools for diagnosing and monitoring livestock and STH infections and, more broadly, backed by a greater understanding of the value of molecular diagnostics in tracking transmission and variants of concern during the pandemic. One benefit of COVID-19 may be the incentive to speed up the implementation of molecular tools in a range of different settings. We would like to point out that our identification of the S168T variant in *Haemonchus contortus* in 2022 has since been developed into LAMP and amplicon-based diagnostic tools and validated in several resistant populations worldwide. So there is both an academic and translational aspect to the data and resources we provide, both founded in the longer term aim of “inform[ing] more effective and sustainable control”.

Reviewer #2: (No Response)

Reviewer #3: I have no major issues.

**Part III – Minor Issues: Editorial and Data Presentation Modifications**

Reviewer #1:

Line 40-41 (abstract): reads “most important pathogens of [sheep and goat] farming”, which implies that T. circ is a pathogen of farming. Suggest delete “farming” from this sentence to read simply “most important pathogens of sheep and goats”. This is repeated in lines 119-120 in the main text.

**RESPONSE:**

Good point. Corrected as suggested.

Line 572: Would “concurrent” be more correct? According to the online Merriam-Webster Dictionary, intercurrent is a term used in pathology in reference to a second, unrelated, disease that occurs during the course of a first or initial condition. The authors mean, I think, combination anthelmintic therapies. Why not say that BZ’s are used frequently in combination formulations with ML’s? That is what happens. Or do the authors mean that IVM (and/or pther ML’s) are used primarily when BZ’s fail? Or simply that since BZ’s preceded IVM into the market by two decades, extensive selection for BZ resistance had occurred before IVM was introduced?

**RESPONSE:**

Thank you for bringing this point up. In the UK, the use of combination products is very limited - we have flukicide/nematocide products which combine anthelmintics, however, we do not, to my knowledge, have products which combine two solely nematode anthelmintics (<https://www.scops.org.uk/internal-parasites/scops-know-your-anthelmintics-guide/>). We meant intercurrent, as farmers here often use different anthelmintics sequentially over the season or in different years. Indeed, this is often advised.

However, we agree that this should be applicable to a global audience and have included a sentence which acknowledges the use of combination products:

*“In some parts of the world, combination products containing more than one nematode anthelmintic are used.”*

Reviewer #2: (No Response)

Reviewer #3: I have only three minor issues.
1 --- The comparisons in Figure 2 are difficult to interpret. I had to go back and forth between the text and the figure, which made the interpretations more difficult. I suggest that the colors for different chromosomes can be dropped. They do not add any meaning that is not already conveyed by the plotting in separate boxes for linkage groups. I suggest that colors could be used to show which drugs were used in the different isolates in the comparisons. If that ends up being too confusing, then maybe expanding the facet labels would be helpful.

**RESPONSE:**

We agree that Figure 2 is complicated and describes a huge amount of data and evolutionary history between isolates. It aims to give a broad, zoomed-out sense of the genetic structure of the isolates and evidence of peaks of differentiation associated with treatment responses. It is not intended for details, although we provide two descriptions of these data, one from a genome-wide perspective and the other from a drug-specific standpoint.

The colours are used to be consistent with Figure 1. Removing them does not help improve the interpretation but does diminish the comparison of datasets between figures.

Similarly, colouring by drug use doesn't quite work, as some are directly exposed to drugs, whereas others are phenotypically resistant, following previous anthelmintic use over time. Moreover, some strains are multi-drug resistant, which would further complicate the colour scheme.

In response to your comment, we have changed the figure and placed the label for the comparison at the top of each plot. This was the only way to make the text more prominent to help the reader, and we think it works well. We have also removed the chromosome boxes and simplified the chromosome names, which makes the data stand out better.

2 --- How many beta-tubulin genes are found in this new assembly? As seen in other nematode species, the composition of beta-tubulin can influence benzimidazole resistance. These data could just be an interesting point to add along with the beautiful connections to putative isotype 1 and isotype 2 variation in Teladorsagia circumcincta BZ resistant isolates.

**RESPONSE:**

This is a good question which forced us to find out. In response to this question, we analysed the genome assembly using the canonical isotype 1 sequence as the query for exonerate. We found five copies of beta-tubulin in the genome: one on chromosome 1 (isotype 1), two on chromosome 2 (isotype 2 and one other, approximately 300 kb away), and two on the X chromosome. As a sanity check, using the same approach, we also found five copies in the WASHU genome assembly, which is reported in the Choi et al. (WASHU) draft genome paper.

We have now included this information in the text.

*“Five beta-tubulin genes were identified in total in the tci2_wsi3.0 assembly, although not all have been associated with resistance previously.”*

3 --- The authors state that moxidectin and ivermectin are similar, which they are as MLs. However, the Kaplan lab has shown that IVM resistance and MOX resistance are likely different because ordered treatments lead to different results in H. contortus. Milbemycins like MOX can circumvent IVM resistance for a period of time before general ML resistance is detected by FECR and LDA. The section on MOX just needs some slight edits to clarify this point.

**RESPONSE:**

Thank you for your comment. We agree entirely that resistance may vary between ivermectin and moxidectin and had previously suggested this in the results section where we had said:

*“Moxidectin and ivermectin may share molecular targets, however, the genetic response to selection by each drug on those targets may differ. This could, in part, explain some differences in MTci2 comparisons with MTci7 relative to ivermectin-resistant isolates, such as the absence of the ivermectin peak on chromosome 5 or the more prominent peaks on chromosomes 4 (peak range: 85-88 Mb) and 5 (peak range: 73-76 Mb) in MTci2:MTci7 relative to MTci2:MTci5.”*

We also pointed out differences in the *pgp-9* copy number (as estimated by the ratio of read coverage depth over this gene between samples) between MTci7 (multi-drug resistant, including both moxidectin and ivermectin) and MTci5 (multi-drug resistant but without adult resistance to a full dose of moxidectin) compared to MTci2.

We discussed differences in the phenotypic development of resistance of moxidectin vs ivermectin:

*“Moxidectin is a macrocyclic lactone and belongs to the same drug class as ivermectin. Phenotypically, resistance to moxidectin typically confers resistance to ivermectin; however, resistance to moxidectin may occur independently of ivermectin resistance in C. elegans* [*(Brinzer, Winter, and Page 2024)*](https://paperpile.com/c/SRKqLV/jqImW)*, a phenomenon also found in a recent field study of gastrointestinal nematodes on sheep farms* [*(Bassetto et al. 2024)*](https://paperpile.com/c/SRKqLV/kKZVh)*.”*

However, we had not pointed out that moxidectin usually continues to have efficacy following ivermectin resistance in the field, and have added a sentence to this effect:

*“ More commonly, however, moxidectin can still be used to control nematode parasites in ruminants following a diagnosis of ivermectin resistance for a period of time* [*(R. M. Kaplan et al. 2007)*](https://paperpile.com/c/SRKqLV/xJEF)*, which may be due either to increased potency (a higher lipophilicity) or a different mechanism/inheritance of resistance* [*(Prichard, Ménez, and Lespine 2012)*](https://paperpile.com/c/SRKqLV/Kf4Y)*.”*

REVIEWER 2 COMMENTS:

The importance of contiguous genome assembly for the accurate interpretation of genome-wide genetic data has already been well established by the authors and others in the parasite genomics field. Perhaps a more nuanced and critical takeaway from this work is that, while a high-quality reference assembly is important, sufficiently resolving drug resistance loci to identify genetic markers remains a significant challenge. Although the FST peaks were linked to known resistance genes in the region, it appears that the resolution of the association signal was not sufficient to *de novo*identify any of the established, let alone novel, anthelmintic resistance genes from the genomic data. This observation does not necessarily diminish the value of the present work but rather highlights an opportunity to explore the various other factors (such as differences in mapping approaches, sampling, and genotyping methods) that influence the resolution of genome-wide studies aimed at identifying resistance loci that can serve as diagnostic markers. A more in-depth discussion of these factors would offer valuable insights and serve as guidelines for future studies and experimental designs in *T. circumcincta* and other related species.

**RESPONSE:**

Thank you for your comment. We have discussed the benefits and limitations of the various experiments used in this study throughout the paper, where information has been relevant. We have added these sentences following a paragraph in the discussion which had been dedicated to these experimental considerations:

*“Although working with highly heterozygous populations, which are sexually reproducing obligate parasites, and which yield very low quantities of DNA per worm (e.g. < 1 ng per L3, the most accessible free-living stage), will always be a challenge, the reducing cost of amplicon sequencing and improved methods for sequencing ultra-low input DNA* [*(Doyle, Sankaranarayanan, et al. 2019; Stevens et al. 2023)*](https://paperpile.com/c/SRKqLV/TGwC+fkXXH) *provides scope to perform individual sequencing of many more individuals than were used in the ddRAD-Seq experiment here. Such work may assist in narrowing our anthelmintic resistance loci. The new genome assembly also allows for older techniques, such as microsatellites, which were successful in narrowing down ivermectin resistant to chromosome 5 in H. contortus* [*(Redman et al. 2012)*](https://paperpile.com/c/SRKqLV/0v4tt) *and an artemisinin resistance marker on chromosome 13 of Plasmodium falciparum* [*(Cheeseman et al. 2012)*](https://paperpile.com/c/SRKqLV/WiXT)*, to be reconsidered, as the relative position of each microsatellite within the genome can now be known. These may provide a more universal scope for investigating resistance where sequencing resources or funds are limited. ”*

Line 45: “573 Mb assembly (N50 = 84 Mb, n = 1,286)”. The "n" represents the total number of scaffolds in the assembly, not the N50 number. To clearly convey this, please consider revising it to “scaffold n = 1,286.”

**RESPONSE:**

Good spot. We intentionally used “n” rather than “N50n” as we wanted to refer to total scaffolds.

In response to this comment, we have added “total scaffolds” to make this clearer.

Line 159: “genome assembly for the UK-derived MTci2 isolate of *T. circumcincta* (tci2_wsi3.0; Table 1)”. Please provide the assembly accession here or elsewhere in the paper.

**RESPONSE:**

Apologies. We hoped to have this submitted to WormBase ParaSite in the last version release; however, it wasn’t.

In response to this comment, we have submitted it to ENA, and the accession numbers are described throughout where appropriate. We have also made clear the following in the data availability section:

*“The genome and annotation are available under the ENA Project accession PRJEB80725.”*

Line 173: “gene content (Table 1, Supplementary Table 4)”, Based on information in Supplementary Table 4, two different versions of the Tci2 assembly (Tci2_wsi2.4 and Tci2_wsi3.0) were used for the BUSCO analysis, please use only the version described in the paper. BUSCO proteome results should be reported based on the longest isoform per gene for *H. contortus* and *C. elegans*. Compleasm results are likely more accurate than BUSCO ( and were included in Supplementary Table 4), I suggest reporting the compleasm results in Table 1.

**RESPONSE:**

Good spot. There was an intermediate version called Tci2_wsi2.4, however, it was replaced by Tci2_wsi3.0. We can confirm that all analyses, including in the table, were performed using Tci2_wsi3.0. The differences in the table were caused by typos between manuscript versions.

We present the BUSCO proteomes based on the published data and compare them to our assembly. We specifically examined the BUSCO scores of our assembly using the longest isoforms to point out that there is still technical duplication, i.e., errors. We purposefully do not compare H. contortus and C. elegans here, as it is not the comparison we aim to make.

We agree that compleasm is likely more accurate than BUSCO. However, compleasm is not yet suitable for proteomes, and so, for consistency, we have kept both BUSCO results in Table 1. Irrespective of accuracy, there is sufficient variation in the output of BUSCO software versions that, in reality, comparisons among BUSCO scores can only reliably be performed within an analysis using the exact same parameters. This is what we have done here.

Line 174: “repetitive content (Supplementary Table 5)”. Assembly names are not consistent across the text, tables, figures, and supplementary material.

**RESPONSE:**

We agree; thanks for pointing this out. In response to the comment, we have made the assembly names consistent throughout.

Line 244: “Comparison of genetic differentiation between pre- and post-treatment on Farm 1 (Figure 2A; top panel) and Farm 2 (Figure 2A; middle panel) both revealed...” The figure legend indicates that the dashed lines in Figure 2A represent a genome-wide level of significance (FST = 0.0339). This value was determined based on the mean + 5 standard deviations of the genome-wide FST calculated from technical replicates of the Farm 2 pre-treatment group (Supplementary Figure 2).

“DNA from Farm 2 samples was split into two sub-samples (“A” and “B” samples), from which individual libraries were generated and sequencing performed.” It is not clear when the samples were pooled. Did you pool the L3 larvae prior to DNA extraction, or did you pool equimolar DNA prepared separately from each individual larvae? Did the "A" and "B" samples come from the same group of larvae, or from two distinct groups of larvae sampled from the same population? To empirically determine the outlier threshold using replicates, one would need distinct pools of larvae sampled from the same population. Additionally, the measured variance of FST depends on both, number of individuals in the pool and the total sequencing depth of each pool, and the extent of deviation from the equimolar assumption between individuals. These variables need to be kept constant across all pools included in the analysis to use a single threshold value across multiple comparisons between different pools. Essential information about the experimental design (e.g., number of individuals, sequencing depth for each pool) is missing, making it difficult to assess whether the FST distribution in the replicates adequately represents the null distribution expected in the absence of selection.

**RESPONSE:**

As described in the methods section, the “A” and “B” samples are technical replicates. Genomic DNA from individual larvae were pooled independently in approximately equimolar proportions. Given that they are a technical replicate of the same group of larvae, variation between them is technical rather than biological variation, including variation associated with selection. We agree that sampling from different groups of larvae from the same population would provide a more accurate estimate of the biological variation of sampling two distinct groups; however, we did not do this and, again, do describe it clearly as a technical replicate. We note that these A and B samples were used to calculate an outlier threshold only for the Farm samples, which were all sequenced together and generated a consistent amount of data. The number of individuals per pool is reported in the methods - 91 per pool.

Line 246: “peak of differentiation”. Starting from this paragraph, the distribution of FST outlier regions (peaks) identified from various mapping experiments are described. However, these peaks are not clearly and consistently defined. The genome-wide significance level for outlier region was defined as 0.0339 for the farm data and as the mean + 3 standard deviations for the strain comparisons. However, no threshold was applied to the peaks in the Choi data. Using a common threshold definition across all datasets would make the comparison of peaks across datasets easier and may more clearly highlight the distinct features of each dataset using different mapping approaches. In addition, not all sliding windows with high FST values above the significance threshold were considered to represent peaks, to account for noise in the data. However, it was not clearly defined how many adjacent or closely spaced outlier windows were required to consider the region as a peak. This lack of clear definition resulted in suggestive language throughout the text, such as “a possible peak,” “may be shared by,” “potential peak,” “suspected peak,” etc. After defining the outlier regions (peaks), they could perhaps be labelled in the figure and referenced in the text. This would greatly enhance readability, given the number of comparisons and peaks discussed in this and the following paragraphs. Finally, please consider including the FST values, their Z-scores, the number of variant sites used to calculate the mean FST, and the read coverage breadth for the individual sliding windows in the supplementary material.

**RESPONSE:**

We agree that a common threshold would make a comparison of peaks easier. However, the experiments and data are so very different that a common threshold does not make sense. This is made clear based on the very different y-axis scales between Farm, Choi, and Strain datasets. It is not at all surprising that the distribution of Fst values is very different between datasets - as we describe in the text, they assess selection at different timescales, from immediate (Farm) to many parasite generations (Strain), directly (adults/L4, treated) and indirectly (L3 or untreated) and therefore there are very different levels of genetic diversity within and between comparisons.

No threshold was applied to the Choi dataset as none made sense given the elevated Fst throughout the genome. The data is simply too noisy.

In response to this comment, we have provided Fst values and variant sites per group in Supplementary Tables 13, 14, and 15. Given that the analysis is pairwise between all combinations of samples within a group, coverage does not make sense to report. However, per the methods and code, coverage limits were set at a minimum of 10 and a maximum of 100, based on comparing the median coverage of samples before running the Fst analyses.

Line 247: “suggesting a common genetic response to ivermectin treatment.” Cochran-Mantel-Haenszel (CMH) test can be performed on the combined Farm1/Farm2 dataset to identify the common genetic response to IVM treatment and differentiate it from farm-specific variation in a more statistically principled manner.

**RESPONSE:**

Thank you for suggesting this. We have now performed a CMH test using the UK farm samples, including the Farm 1 pre- vs post-treatment pairwise comparison, and the Farm 2A technical replicate pre- vs post-treatment comparison. This has further accentuated the ivermectin locus under selection, but applying a false discovery rate, or the Bonferroni correction identified only a maximum of two SNPs, one of which is just peripheral to the locus. We expect that, although it is certainly useful to further corroborate the region as being under selection, it may be underpowered to identify individual SNPs driving ivermectin resistance in these populations. Alternatively, there may be different SNPs independently selected on each farm.

We have added the following to the methods:

*“To identify whether any SNP alleles were under selection by ivermectin on both UK farms, a Cochran-Mantel-Haenszel test was performed using PoPoolation 2 (v1201, “popoolation2-code” version which provided the log odds ratio) cmh-test.pl (--min-count 3 --min-coverage 10 --max-coverage 2% --population 1-2,3-5). The Farm 1 pre- and post-treatment pairwise comparison was compared with the Farm 2A pre- and post-treatment pairwise comparison. P values were filtered using both the FDR (Benjamini-Hochberg), choosing an FDR of 5%, 1% and 0.01%, and also by using the Bonferroni method. Finally, the proportion of SNPs ranked within the top 1% of the lowest p-values, which also had a probability of a reduction in the reference allele in the post-treatment samples relative to the pre-treatment samples (log odds ratio < 1), were calculated in 1 Mb windows along the chromosomes and plotted using ggplot2.”*

And to the results:

*“To search for SNP alleles under selection by ivermectin on both farms, a Cochran-Mantel-Haenszel test was performed, using the Farm 2A technical replicate comparison. This accentuated the significance of the 36-38 Mb region in particular (Supplementary Figure xxx), however only two SNPs were identified (Benjamini-Hochberg FDR 5%) as being significant following correction for multiple tests; Chr 5:38,037,930, peripheral to the ivermectin locus as defined here, and Chr 5:36,505,260, predicted to be an intron variant of TCIR_00147810, an orthologue of a H. contortus G protein receptor domain containing protein. Given the availability of only two pairwise comparisons for the CMH test, we would advise that additional tests are performed to consider whether particular SNPs are important in ivermectin resistance, and whether multiple variants may individually (as for benzimidazoles or monepantel), or collectively confer ivermectin resistance.”*

And we have placed two figures in the supplementary file.

Line 251: “Comparison of the post-treatment populations from Farm 1 and Farm 2 revealed a more complex history of genetic change and likely drug-mediated selection”. Distinguishing the effects of drift and selection in between-farm comparisons is challenging. However, comparing the pre-treatment populations from Farm 1 and Farm 2 could provide a clearer insight into historical genetic changes, as opposed to the post-treatment population comparisons presented in the paper, which are confounded by the direct effects of selection.

**RESPONSE:**

We agree that it can be difficult to distinguish between drift and selection in the between farm analyses. We had previously compared pre-vs-pre treatment between Farm 1 and Farm 2 and didn’t feel that it provided any new insight into interpreting the data relative to what we have already shown. However, for transparency, in response to the comment, we now show the comparison in Supplementary Figure 4 and refer to it in the text as follows:

*Supplementary Figure 4. Distinguishing genetic drift from selection on the UK Farm populations.*

*The two plots compare Farm 1 post-treatment vs. Farm 2 post-treatment (top; as shown in Figure 2A) and Farm 1 pre-treatment vs. Farm 2 pre-treatment (bottom). These data support the hypothesis that most of the genetic differences between farms existed before the most recent treatment, as shown in the comparisons of pre- vs. post-treatment in Figure 2A, and that the chromosome 5 peak is treatment-specific.*

Line 297: “Comparison across all three panels reveals common peaks of differentiation”. CMH test could be useful for identifying and statistically supporting common genetic differences across multiple pairs of pools.

**RESPONSE:**

Please see above comment regarding CMH test performed for the ivermectin locus. We have only two pairwise comparisons (farm data) we could use for a CMH test as the others are all individually different experiments.

Line 320: Figure 2. The assembly name could be removed from the chromosome ID. The life cycle stage of the samples used in the Figure 2C comparisons was not provided.

**RESPONSE:**

We agree that the assembly name is unnecessary in the figure and have removed it as suggested.

The lifecycle stage of the samples used in Figure 2C is provided in Supplementary Table 2. To make it clearer, we have now included in the figure legend:

*“Sample details including life stage composition is described in Supplementary Table 2.”*

Line 340: “We have compiled a list of candidate genes associated with individual drug classes and previously described variants associated with resistance (Supplementary Table 7).” Please indicate which of these candidate genes were contained within the FST peaks identified in this study and provide references for the previously described variants associated with resistance. The resistant allele frequencies and the statistical significance of the frequency differences (from the relevant pairwise comparisons) should be included.

**RESPONSE:**

We have already made a conscious effort to report candidate genes that overlap with the peaks of differentiation in the text. However, we have now expanded on this and other details regarding genes in peaks in Table 2.

In the results, we have already provided references for the previously described variants associated with resistance in the benzimidazole and levamisole sections.

We provide the resistant allele frequencies for known variants for benzimidazole and levamisole in the text and for benzimidazole in Figure 3. A statistical test for frequency differences has not been performed, as we do not have the appropriate replication to perform a test. The different comparisons are more or less independent of the different strains compared, so the data is descriptive.

Line 355: “we identify the F200Y allele at high frequency in all strongly resistant isolates, as well as the E198L variant”. Choi et al. (2017) reported that the F200Y and E198L variants likely occur only in trans on separate haplotypes in their study population from New Zealand. (minor comment, introduce earlier than the discussion section in the manuscript that the 2017 study is based on New Zealand strain). They concluded that the absence of haplotypes simultaneously harbouring both variants suggests that E198L can confer BZ resistance independently of F200Y and that beta-tubulin carrying both variants is either detrimental to organismal fitness or that the double mutation event (or recombination over an interval of <6 bp) required to produce a cis haplotype is so unlikely that it has not been observed. Have you investigated the haplotype structure around the F200Y and E198L variants in the UK samples, particularly in the Farm samples? Could further insights be drawn from these comparisons?

**RESPONSE:**

Apologies, we have now mentioned that the Choi strains are from New Zealand in the introduction and results sections.

As previously mentioned in response to reviewer 1, we did not do any haplotype analysis of beta-tubulin variants as it was already clearly established that F200Y and E198L variants could only occur in trans and not in cis, explaining why we do not see homozygous resistant double variants. However, in response to this and the reviewer 1 comment, we did examine haplotype frequencies in all samples. Consistent with the results of Choi et al., we do not find double homozygous variants in the same haplotype, and individuals carrying both resistant variants must be doing so in trans as heterozygotes.

In response to this comment, we have new data in Supplementary Table 8 and have added the following text:

*“ Consistent with Choi et al., we did not find any haplotypes containing both resistant alleles for both E198L and F200Y in any pooled sequencing dataset (Supplementary Table 8), further supporting the hypothesis that heterozygotes for both alleles are in trans- rather than cis- and that individuals homozygous for both resistance alleles are unviable* [*(Choi et al. 2017*](https://paperpile.com/c/SRKqLV/s0nnh)*).”*

Choi et al. also noted that F200Y occurs on at least two distinct and diverse haplotype backgrounds, suggesting multiple independent origins of the variant allele. When multiple distinct haplotypes are under selection, soft sweep-like signatures may be observed around the loci. In these regions, individual SNP-level (FST or allele frequency) comparisons can provide stronger and more meaningful signals compared to sliding window analysis. For example, individual SNP-level analysis within the major ivermectin peak (Chr 5) may provide additional resolution to narrow down the candidate loci.

**RESPONSE:**

We intentionally did not do individual SNP-level analyses, as we are not powered to make robust inferences to discriminate linked from causal variation. We consciously decided to exclude these data, as they might mislead the reader and the research field by reporting variants that are statistically variable but likely meaningless.

Instead, we focused on describing the genomic landscape, which is a valuable first step towards defining meaningful genes and variants. In doing so, we have prioritised known or proposed candidate genes based on their position relative to peaks of differentiation and excluded those in regions of the genome that are not associated with resistance.

Pool-seq data enables the analysis of within-population genetic diversity, such as pi. Comparing pi between pre- and post-treatment samples can offer valuable insights into the extent of genetic diversity loss driven by selection and, consequently, the nature of that selection. Have you observed a pronounced decrease in genetic diversity within the identified FST peaks following treatment? These analyses could provide complementary evidence for the resistance loci, particularly in the novel regions.

**RESPONSE:**

This is a logical suggestion - we did measure Pi in all populations, and while we do see a greater loss in genetic diversity in regions we propose are under selection, it is more subtle than our Fst analyses. See the example below, comparing pre and post-treatment populations on Farm 1 and 2. In our Fst analyses, we identify a peak of differentiation at approximately 37 Mb on chromosome 5. Here, we see a greater loss of genetic diversity in the post-treatment populations of both farms relative to their pre-treatment populations. However, it is harder to see and requires the reader to know what they are looking for. We feel this adds little value and may confuse the reader, so we have decided not to include it.

Line 372: Figure 3. Please revise the X-axis title. "Sampling location" is appropriate only for the Farm samples. Include the statical significance of the frequency differences (e.g., Fisher’s exact test using allele counts).

**RESPONSE:**

We agree that the x-axis title “Sampling location” is inappropriate for all sample groups and have removed it from the figure, as suggested.

We are simply describing the allele frequencies here; there is no pairwise comparison to do a statistical test.

Line 417: “A single peak on chromosome 3 (peak range: 15-20 Mb) found only in the MTci7:MTci2 comparison and not in the other ivermectin-resistant isolate comparisons is suggestive of a moxidectin-specific response.” As discussed in my previous comment (Line 246), because FST peaks are not clearly defined, it is difficult to determine whether the peak on Chr 3 (15-20 Mb) is truly a moxidectin-specific response region and whether there are other, potentially more significant peaks.

**RESPONSE:**

Thank you for your comment. We refer to the Chromosome 3 15-20 Mb peak as a moxidectin-specific peak, not because of its height or defined boundaries but because it has only occurred in the MTci7:MTci2 comparison, the only isolate known to be moxidectin resistant in our study, and we remark that is it “suggestive of a moxidectin-specific response”, as we have only the single comparison to test this. We respectfully suggest that whether a peak is strongly and clearly defined is relative to the analysis being undertaken - comparisons of the benzimidazole locus, for example, tend to always result in very clear and tall peaks, particularly post-treatment. While other comparisons can result in broader or shallower peaks. Noise in the data due to diversity unrelated to anthelmintic resistance can also make peaks less clear. Nevertheless, we would argue that the Chr 3 peak is clearly visible, and thus, we define it as a peak.

Line 438: “compared Fst from pooled sequencing (Figure 4A) with an independent experiment using ddRAD-seq”. It is not clear what is meant by “independent experiment.” Line 942 states, “These L3 were a subset of those included within the larger number pooled for whole-genome sequencing above.” Clarification is needed.

**RESPONSE:**

Thank you for asking for clarification. We had meant a different kind of experiment to the pool-seq. In the methods we had previously written

*“These L3 were a subset of those included within the larger number pooled for whole genome sequencing above.”*

However, to make it clearer that this was so, we have slightly altered the line in the results to now read:

*“To explore this region further, we focused on the Farm 1 population, in which we compared Fst from pooled sequencing (Figure 4A) with an independent experiment using ddRAD-seq to assess linkage disequilibrium (LD) among genotypes from individually re-sequenced larvae from the same pre- and post-treatment sample populations (Figure 4B).”*

Line 439: “ddRAD-seq to assess linkage disequilibrium (LD)...”. LD decay analysis (Fig 4B) is likely sensitive to variations in missing genotype calls and allelic dropouts between the pre- and post-treatment populations. Have you confirmed that the differences in the decay patterns were not associated with any nonrandom genotype missingness in the ddRAD-seq dataset? Did you observe an FST peak in the ddRAD-seq dataset comparable to that from the pool-seq analysis? Was the density and distribution of ddRAD loci adequate for reliably measuring the rate of LD decay at a sufficiently high resolution? Because continuous line graphs were used, and decay distance at a single r^2 value was plotted, it is difficult to determine how robust and well-supported the signal is. An alternative approach that visualizes all genotyped locations and all pairwise LD values within the region (e.g., PMID: 33126247) may help readers better assess the significance of the LD variation between pre- and post-treatment populations.

**RESPONSE:**

We agree that analyses can be affected by the depth of coverage (“sensitive to variations in missing genotype calls and allelic dropouts between the pre- and post-treatment populations”). Similarly, we recognise your point regarding whether the density and distribution of RADloci were sufficient for the investigation. For this reason, after calling variants, we first checked the individuals for missingness and subsequently excluded two individuals previously identified as being identical in genotype (likely a technical error in sample preparation as they were prepared at the same time) and pointed the reader to a thesis containing further information on this analysis. “A prior analysis using an earlier version of the assembly had identified that two individuals – one in the pre-ivermectin sample and the other in the post-ivermectin sample – were identical, and thus, these individuals were removed from further analysis (see [(J. R. McIntyre 2020)](https://paperpile.com/c/SRKqLV/o2LWN) for more details).”

To ensure that RADloci were present in sufficient individuals, we simultaneously took into account (1) the high nucleotide diversity expected between individuals in *T. circumcincta* populations, which could lead to RADloci drop out, (2) the expectation that RADloci under selection should be present in both the pre-and post-treatment populations, even with a moderately small sample size (n=29 pre, 31 post, see methods) and that (3) RADloci which might be present predominantly because of a variant under selection (by linkage or direct selection) should be present in about half or more of individuals post-treatment in our experiment. As we had written in the methods:

*“The vcf was filtered using GATK VariantFiltration to keep only variants successfully genotyped in at least 30 individuals (--filter-expression “AN < 60”) and with a mapping quality greater than 30 (--filter-expression “MQ < 30”). A threshold of 30 individuals was chosen to include RAD sites, which were present in the pre-treatment or the post-treatment sample population but lost in the other.”*

RADloci retained in the vcf file would be common but allowed for RADloci to be directly under selection and therefore greater in only one of the samples, as LD was calculated within-sample, not between samples.

For calculations of r2, 75% of individuals within a sample had to contribute to the locus, and the minor allele frequency required that at least two or three individuals encoded the variant, dependent on the sample (see methods). Regarding the density of loci across the genome and the locus, we describe in the methods that we thinned variants used to 1/100 kb for inter-chromosomal r2 (used to identify ‘background’ LD) and to 1/1 kb for chromosome 5. We had previously plotted the individual data points, visualising the LD over the locus. However, we felt that the line plots were clearer and more easily interpretable to readers while displaying the same information. As we have stated in the results, we did not have sufficient RAD loci over the 34-35 Mb region to exclude that this region of chromosome 5 was not under selection. We have now made it clear that this was the RADloci specifically:

*“although due to lower coverage of RADloci between 34 and 35 Mb, this region cannot be excluded”.*

Although we could not exclude it, we tested whether it extended through and beyond it (i.e. was there LD between 32/33Mb and 35/36 Mb) and found that it did not. We are confident that the decay pattern differences are associated with ivermectin resistance as they correlate with the Fst Pool-seq plots in both the farms and the MTci5(IVM):MTci2 data. However, other decay pattern changes were seen over chromosome 5, as described in the results, which we believe are not associated with ivermectin resistance due to a lack of peaks in Fst data. One of these, we do not understand its cause, the other we identify as showing an Fst peak when comparing farms and isolates.

The line graphs do not show the decay distance at a single r2 value but rather the decay distance to that r2 value. This was calculated in distinct sliding windows along the genome, using a script to calculate the decay distance to the mean inter-chromosomal r2 value (0.05) within each window. This distance was then plotted. It, therefore, used all the RADloci data and provided a single distance value to plot. Where the linkage decay was less (LD was greater), the distance at which r2 was still equal to 0.05 was longer, so the peak was higher.

The GitHub code provided with the paper provides a detailed explanation of the quality checks done, including plots to assess RADloci density.

We did not calculate Fst for the RADloci data as they were generated from a subsample of the same individuals used for the Pool-seq, and we were using them for a different experiment—to identify the extent of linkage disequilibrium within the region.

Line 442: “consistent with a reduction in diversity associated with drug-mediated selection”. Sliding window analysis of pi using the pool-seq and/or ddRAD-seq data would show the reduction in diversity in the region.

**RESPONSE:**

Please see the above comment about the use of Pi.

Line 451: “variation in coverage was noted”. Coverage ratio variation (at a 100 kb window size resolution) in Fig 4C likely indicates copy number variation (CNV) within the FST peak. The maximum ratio (~1.5) could be an underestimation if the size of the CNV is relatively small compared to the window size. Using a dedicated CNV caller (e.g., PMID: 22302147) rather than relying on a heuristic approach would improve both the sensitivity and specificity of CNV detection and may identify resistance-associated CNVs.

**RESPONSE:**

We agree that calculating coverage in a 100 kb sliding window is an imprecise way to measure coverage, especially, as the reviewer points out, if the CNV is much smaller than the window. This was not the point of this comparison for relative coverage, i.e., to define CNVs specifically, but to see if there was a bias in the Fst due to some differences in coverage. We feel that in addition to CNV, there is the possibility that increased diversity within a sample relative to the reference genome could result in reduced read alignment.

As the reviewer has found further on, we did look at gene-based CNV variation, particularly in the context of the previously described copy number variation of pgp-9. We believe these are more robust comparisons of CNVs compared with a genome-wide approach, as the mapping of sequencing reads to exons is more reliable and less variable relative to intronic and intergenic regions. We did not find CNV-related genes in our gene-based CNV approach lying within the region of chromosome 5 of interest and, hence, do not believe further analysis will reveal anything new.

Line 455: “These results collectively suggest that the locus under ivermectin selection lies between 35-38 Mb”. Conducting the CMH test across all pairwise comparisons that show differentiation signals across the Farm, Choi, and Strain datasets would reduce noise from individual comparisons and could significantly narrow down the regions under direct selection, distinguishing them from linked loci. This approach would highlight the long-term comparability of WGS data across multiple studies and experiments, and the value of the improved assembly, which enables more accurate re-analysis and integrated analysis with previously published data.

**RESPONSE:**

Thank you for the suggestion. We fully agree that this can be a valuable test to compare across replicated experiments, retaining within-experiment variation while also identifying common outcomes. We have performed a CMH test comparing Farm 1 and Farm 2A replicate pairwise comparisons (see above). However, the other experiments are quite different (a genetic backcross (Choi data) and comparison of different isolates (MTci7:MTci2), or different isolates directly following treatment of one (MTci5(IVM):MTci2) and therefore would not be comparable to the farm data. As there are only individual comparisons for each experiment type, we did not feel performing a CMH test with them was suitable.

Line 480-489. These perhaps could be moved to the discussion section.

**RESPONSE:**

We prefer to keep this here, as it provides the context for the analyses we describe and do not come back to it in the same way in the discussion.

Line 504: Supplementary Figure 7. The figure suggests that there are genes likely displaying CNV in each pool-seq comparison. However, since the order of genes along the X-axis varies between comparisons, it is difficult to determine whether any genes other than pgp-9 show CNV across multiple comparisons. Was pgp-9 the only gene consistently detected across multiple comparisons?

**RESPONSE:**

Thank you for the question. It is a good point. We looked specifically for *pgp-9,* as it was previously implicated in MDR related to CNV between resistant and sensitive isolates. While we could look for other genes, we currently have no basis for understanding whether an alteration in their CN would lead to specific resistance. A better experiment would be to examine CNV in individuals in the future to achieve a more robust analysis towards identifying CNV association with treatment responses.

Line 597: “We interpret these more complex signals...” The limited number of backcrosses (n=2) used to generate the RS3 introgressed line in Choi et al paper is likely the primary reason for the broader peak regions observed, rather than the severe bottlenecking that occurs during co-selection. Since sexual recombination occurs before drug treatment, the recombination itself is not directly impacted by bottleneck events. However, the “effective” recombination rate is influenced by the genetic divergence between the two parental lines in the crossing experiment, which can affect mapping resolution. Regardless of the severity of the bottlenecking during selection, as long as there is sufficient genetic divergence between the parental lines and the treatment does not kill all individuals during selection, it would still be possible to achieve high mapping resolution in the introgressed line, provided that a sufficient number of backcrosses are performed.

**RESPONSE:**

We respectfully disagree with the reviewer here. We agree with the reviewer that there were limited backcrosses in the Choi et al. experiments. However, multiple generations of passage with triple anthelmintic treatment were conducted both before (5 generations) and between backcrosses, at unreported population sizes; larvae were passaged, and presumably, a much higher number than adults were sampled at the end or transferred in the backcrosses. With constant treatment with the three drugs simultaneously, only the few parasites resistant to all three drugs will survive.

We can estimate the bottleneck in the Choi cross as follows:

- Let's assume IVM, LEV, and BZ resistance is each controlled by a single gene, with two alleles; one susceptible and one resistant
- The maximum allele frequency of resistant alleles is assumed to be between 0.5 and 1 in the resistant parent, depending on the mode of inheritance (dominant, recessive)
- The frequency of resistant alleles in the susceptible parent is assumed to be 0
- In the cross, the resistant alleles shift to between 0.25 and 0.5
- The frequency of worms surviving treatment of all three drugs is 0.25*0.25*0.25 = 0.016 to 0.5*0.5*0.5 = 0.125, i.e., between 1.6% and 12.5% surviving.
- Then recombination breaks it up, followed by treatment again, further reducing the effective surviving population.
- We know that multiple genes and alleles are involved in resistance to some drugs—these estimates are an upper limit; however, the surviving population is likely smaller still.

This is a severe bottleneck and will result in excessive linkage structure among causal and non-causal variants, including those across chromosomes. Therefore, as we describe in the text, there are complex signals of selection.

The resolution would have been completely different if the original experiment had been performed with one drug rather than three. Our confidence in this is evidenced by our backcross and genomic analysis in *Haemonchus contortus*. In these experiments, four rounds of backcrossing were performed, which is more than two rounds in the Choi et al. experiments. However, the ivermectin-only treatment produced a clear signal specific to chromosome 5, with very little background signal.

Line 645: “Finally, comparing pre and post-ivermectin treatment parasite populations has lowered the background genetic variation commonly observed for genetic comparisons of distinct isolates, allowing identification of a single locus of differentiation. In contrast, between isolate comparisons identified multiple peaks of differentiation which could not be confidently assigned to specific drug classes and may reflect non-drug-related selection and divergence between populations.” Both untreated and drug-treated MTci5 samples were available for comparison. However, the treated MTci5 samples were not compared against the untreated sample. Were those comparisons done, and what was the reason for not including these comparisons in the paper?

**RESPONSE:**

We did, in fact, do the bioinformatic comparisons between untreated MTci5 and ivermectin-treated MTci5 and benzimidazole-treated MTci5. Given that this is a within-strain comparison and that the strain is phenotypically resistant to both ivermectin and benzimidazoles already, no obvious signals are associated with treatment. Therefore, we decided not to include these comparisons as they were not informative. What they may show, which does not add anything to the conclusions already drawn in the manuscript, is that benzimidazole selection occurred further back in time than ivermectin selection as there is less genetic differentiation genome-wide between MTci5(BZ) and MTci5 than MTci5(IVM) and MTci5. This would be as expected given the historical use of these anthelmintics in the UK.

Line 659: “Targeted, fine-scale mapping using samples from drug-specific experiments will likely yield further insight into the specific causal variants driving resistance”. What does “drug-specific experiments” mean in this context? Both Farm and Strain experiments used single drug selection, but it was not possible to prioritize a small number of candidate causal variants from these experiments.

**RESPONSE:**

The Farm experiments analysed selection after administering a single drug, comparing the selection of a subset of a population (offspring from adults surviving treatment) to the whole (offspring from all worms present in the hosts pre-treatment). In contrast, the strain comparisons were made between isolates, and only two used an isolate known to have been treated directly before sampling. All samples had a complex history, some were treated and some were not, and some had historical resistance and selection that did not result from their most recent treatments.

Some more carefully designed “drug-specific” experiments, perhaps involving dedicated sampling and sequencing of larger numbers of worms individually, would likely help unravel drug-specific effects.

A common limitation across the three datasets (Farm, Choi, and Strain) seems to be the difficulty in distinguishing causal loci under direct selection from a large number of linked variants, resulting in broader differentiation peaks. Sequencing individual worms is unlikely to fully address this issue. A paragraph detailing specific approaches and experimental design considerations that may help overcome this problem should be included.

**RESPONSE:**

We agree that it is difficult to distinguish between linked and causal loci in the three datasets. It is one of the key reasons why we have not invested effort in describing specific variants in genes that have no known association with resistance. None of the three datasets are genuinely appropriate for identifying causal variants for specific drug classes. This is perfectly fine, and we have focused instead on describing the genomic landscape of treatment exposure and response and, in some cases, genetic variation in well-defined candidate genes. By understanding the genomic landscape, the design of appropriate experiments to understand further genetic variation in genes in these regions can be now made.

We have tried to be upfront and transparent about the study's limitations (including a specific paragraph on some of these limitations and potential solutions) and purposefully have not overinterpreted our data or included analyses we believe the reader could over-interpret. Hence, we have provided several suggestions for improvements to the experimental design. We have expanded on these to some extent. These include:

- Problem:
  - “We could not fully assign all contig sequences to chromosomes, choosing instead to keep any with at least partial unique sequences as either unplaced scaffolds or assigned to linkage groups”
- Solution:
  - “further improvements could be achieved with investment in single worm genome long-read sequencing, or with ultra-long sequencing reads such as those obtained from Nanopore single molecule sequencing for assembly or further scaffolding”
- Problem:
  - “ivermectin resistance commonly appears on a background of benzimidazole resistance”
- Solution:
  - “the use of a single anthelmintic and comparison of pre and post-treatment samples from an admixed and multi-drug resistant population, as shown here, could help, or via the use of genetic crosses as discussed below “
  - “Further phenotyping of sample populations *in vitro*, with subsequent individual or pooled sequencing, may also serve to distinguish selection signals”
  - “Understanding the genetic relatedness of haplotypes in these regions, particularly from a denser sampling of parasite populations, will further inform how resistance evolves and spreads.”
- Problem:
  - “these and other genomic regions were difficult to interpret using the data from Choi and colleagues independently.”
- Solution:
  - “A similar cross framework followed by drug selection with individual drug classes would better refine the signals to reveal drug-specific peaks. “
- Problem
  - “Additional loci associated with resistance are likely to exist within these data but are difficult to identify using sequencing data from pools of individuals confidently”
- Solution:
  - “it will be important to determine the relevance of these signatures of selection in other drug-resistant *T. circumcincta* from around the world”
- Problem
  - “For monepantel resistance, a variety of mutations, each independently likely to alter the function of *mptl-1,* have been identified in phenotypically resistant *T. circumcincta* populations [(Turnbull et al. 2019)](https://paperpile.com/c/SRKqLV/EPzy), but the diagnostic utility of this information is less clear”
- Solution:
  - “may require an understanding of haplotypes of variants across a larger region; such variants might be monitored by long-read approaches such as PacBio or Nanopore sequencing”
- Problem:
  - “Sequencing of relatively small pools of resistant and susceptible populations to compare genome-wide variation provides a limited level of resolution”
- Solution:
  - “could be refined further by analysing genotypes from large-scale single worm sequencing of phenotypically well-defined populations, albeit at a significantly greater cost than pooled sequencing to measure allele frequencies”
- Problem:
  - “between isolate comparisons identified multiple peaks of differentiation which could not be confidently assigned to specific drug classes and may reflect non-drug-related selection and divergence between populations”
- Solution:
  - “Treatment directly before sampling a population was found to enhance the signal”....
  - “This could be resolved through genetic crosses followed by selection with individual drug classes to focus on drug-specific responses”

Line 699: “sequenced on a single lane of Illumina”. The methods section frequently reports the number of lanes used in sequencing. While this is often done for historical reasons, it is not particularly relevant, as sequencing libraries across multiple lanes and runs on modern platforms do not introduce significant technical bias. Instead, please report the total number of gigabases (Gb) generated per sample, either in the relevant sections or in the supplementary material.

**RESPONSE:**

The number of lanes of a particular sequencing instrument is part of the experimental methods, which is why it is reported. Moreover, it is possible to back-calculate the per-sample data yield from that information, making it particularly relevant.

We have included the number of reads and mapping % in Supplementary Tables 2 and 3 to provide some information on the per-sample datasets.

Line 864: “Protein sequence orthology between genome assemblies was assessed using Orthofinder” and line 867: “Functional annotations for the protein sequences were inferred using InterProScan”. Including a gene mapping between the WASHU and tci2 assemblies and providing the functional annotation results as supplementary material in the paper would enhance the usability of the new genome, particularly for researchers who may lack access to advanced computing resources.

**RESPONSE:**

To further improve the comparisons between genomes and maintain legacy information from the WASHU genome, in response to this comment, we have provided the following information and supplementary tables:

*“To enhance comparison between WASHU and tci2_wsi3.0 assemblies, we provide the orthogroup mapping between predicted protein sequences from the two assemblies in* ***Supplementary Table 11****.”*

And

*“To support further analysis using these data, we provide GO terms associated with tci2_wsi3.0 transcripts in* ***Supplementary Table 12.”***

Using this information, it is possible to quickly map orthologous genes based on the old genome into the new genome, and using the GO terms, gene set enrichment is performed using the new annotation.

Line 920: “All samples consisted of hundreds of adults or L4 worms, sampled before or after drug treatment, as described in Supplementary Table 2”. Please provide the approximate number of individuals pooled and the sequencing depth for each sample. This information is crucial for assessing the quality of the pool-seq experiment, as inadequate sampling can compromise the accuracy of the analysis. Additionally, please report the mapping rates for the pool-seq and RAD-seq data. Anomalous mapping rates may indicate the presence of contaminant DNA in the samples or, in species with high genetic diversity like T. circumcincta, could suggest limitations in read aligners when mapping highly divergent reads to the reference genome. These are important QC metrics that should be included in the Supplementary Table.

**RESPONSE:**

The approximate number is described as hundreds of worms. We didn't count them, as they were archival material in a delicate mass that included broken worms. Had we tried to untangle them, the worms would have broken into many more pieces, making counting them irrelevant.

In our experience, pooling more than tens of worms has little impact on the quality of the experiment's data. Biased sampling would have a greater effect, however, we do not believe this to be the case here.

We have included the amount of data and mapping rates per sample in Supplementary Table 2. We agree that mapping rates can be important but are difficult to appropriately compare and interpret in a study like this in which samples have been collected and data generated at different times and from different strains. Contamination is present (as always in these sample types), and sequence diversity will affect mapping; however, these are confounded and non-independent here and, therefore, cannot be objectively assessed. More important would be to understand whether contaminant sequences remained within the genome. We checked for these when making this assembly and removed contaminant sequences when they were identified. As contaminants generally have a different level of sequencing depth to the species under investigation, they may often be discarded by filtering steps in the analysis.
